# The Oxindole GW-5074 Inhibits JC Polyomavirus Infection and Spread by Antagonizing the MAPK-ERK Signaling Pathway

**DOI:** 10.1128/mbio.03583-22

**Published:** 2023-02-14

**Authors:** Jacob Kaiserman, Bethany A. O’Hara, Kaitlin Garabian, Avraham Lukacher, Sheila A. Haley, Walter J. Atwood

**Affiliations:** a Department of Cell Biology, Biochemistry, and Molecular Biology, Brown University, Providence, Rhode Island, USA; University of Pennsylvania

**Keywords:** GW-5074, JC polyomavirus, PML, cell signalling, experimental therapeutics, nephropathy, oxindole, polyomavirus

## Abstract

JC polyomavirus (JCPyV) is a ubiquitous, double-stranded DNA virus that causes the fatal demyelinating disease progressive multifocal leukoencephalopathy (PML) in immunocompromised patients. Current treatments for PML are limited to immune reconstitution, and no effective antivirals exist. In this report, we show that the oxindole GW-5074 (3-(3,5-dibromo-4-hydroxybenzylidene)-5-iodoindolin-2-one) reduces JCPyV infection in primary and immortalized cells. This compound potently inhibits virus spread, which suggests that it could control infection in PML patients. We demonstrate that GW-5074 inhibits endogenous ERK phosphorylation, and that JCPyV infection in GW-5074-treated cells cannot be rescued with ERK agonists, which indicates that the antiviral mechanism may involve its antagonistic effects on MAPK-ERK signaling. Importantly, GW-5074 exceeds thresholds of common pharmacological parameters that identify promising compounds for further development. This MAPK-ERK antagonist warrants further investigation as a potential treatment for PML.

## INTRODUCTION

JC Polyomavirus (JCPyV) is a small, double-stranded DNA virus that is the causative agent of the fatal demyelinating disease progressive multifocal leukoencephalopathy (PML) (1–4). Infection is ubiquitous, as between 60 and 80% of adults are seropositive for JCPyV (3, 5–7). JCPyV is thought to persist in the kidneys, and asymptomatic individuals secrete virus in the urine (1, 2). Under immunosuppressive or immunomodulatory conditions, JCPyV invades the central nervous system (CNS), and infects oligodendrocytes and astrocytes (1–3). Lysis of the myelin-producing oligodendrocytes leads to white matter lesions and symptoms characteristic of PML (1–4). While the incidence of PML is rare, prognosis of patients affected with PML is extremely poor, as PML is associated with a 30 to 50% mortality in the first few months after diagnosis, and patients that survive are often left with permanent neurological deficits (1–4).

PML has been traditionally associated with HIV-AIDS, hematological malignancies, or treatment with immunomodulatory drugs including Tysabri (Natalizumab) (1–3). Current treatment guidelines focus on removing the cause of the underlying immunosuppression, and in recent years, antiretroviral therapies and extended-interval dosing have effectively reduced the incidence and severity of PML in these primary populations (1–4, 8–11). Despite these successful management strategies, immunological and epidemiological obstacles still exist. Reestablishment of the immune system is associated with immune reconstitution inflammatory syndrome (IRIS), a hyperactive immune response that occurs in up to 18% of HIV-associated PML and nearly 70% of patients with natalizumab-associated PML (11–13). PML-IRIS is often managed with high-dose glucocorticosteroids that dampen the overactive immune response, but simultaneously antagonize a patient’s ability to fight JCPyV (3, 12, 13). In total, PML-IRIS is associated with a 28% mortality rate (11–13). While the incidence of HIV- or natalizumab-associated PML has decreased, new risk groups are emerging, as reports estimate that the number of autoimmune diseases associated with PML has increased since 2006 (11). This increase can likely be attributed to new immunomodulatory agents including fingolimod or resiniferatoxin (11, 14, 15). As such, more research is needed to identify viable treatment strategies for PML, because there are no antiviral therapies or prophylactic vaccines for this disease.

Additionally, JCPyV is closely related to other polyomaviruses that cause human disease following immunosuppression including BK Polyomavirus (BKPyV). BKPyV is the causative agent of hemorrhagic cystitis, a diffuse inflammation of the bladder that affects between 5% and 20% of bone marrow transplant recipients, and BKPyV-associated nephropathy, a common complication of kidney transplants that leads to graft rejection in approximately 50% of cases (8, 16–18). Like PML, effective treatment options for these BKPyV-associated morbidities are lacking (8, 16–18).

In this report, we identify the C-Raf inhibitor GW-5074 (3-(3,5-dibromo-4-hydroxybenzylidene)-5-iodoindolin-2-one) as a novel antiviral agent. We show that this compound reduces initial JCPyV infection in immortalized and primary astrocytes. GW-5074 also potently inhibits viral spread in an established infection, which suggests that it could control infection *in vivo.* Treatment with GW-5074 drastically reduced endogenous ERK phosphorylation, and JCPyV infection could not be rescued in cells treated with GW-5074 and ERK agonists. These data suggest that GW-5074 exerts its antiviral activity by antagonizing the MAPK-ERK signaling cascade. Finally, we calculate that the minimum 50% selectivity index (SI_50_) associated with GW-5074 treatment exceeds the SI_50_ values associated with many compounds with known anti-JCPyV activity, including mefloquine, chlorpromazine, and Retro-2^cycl^. These findings establish the therapeutic potential of GW-5074 as a novel treatment or prophylaxis for PML.

## RESULTS

### GW-5074 reduces initial JCPyV infection in glial cells.

**FIG 1:**
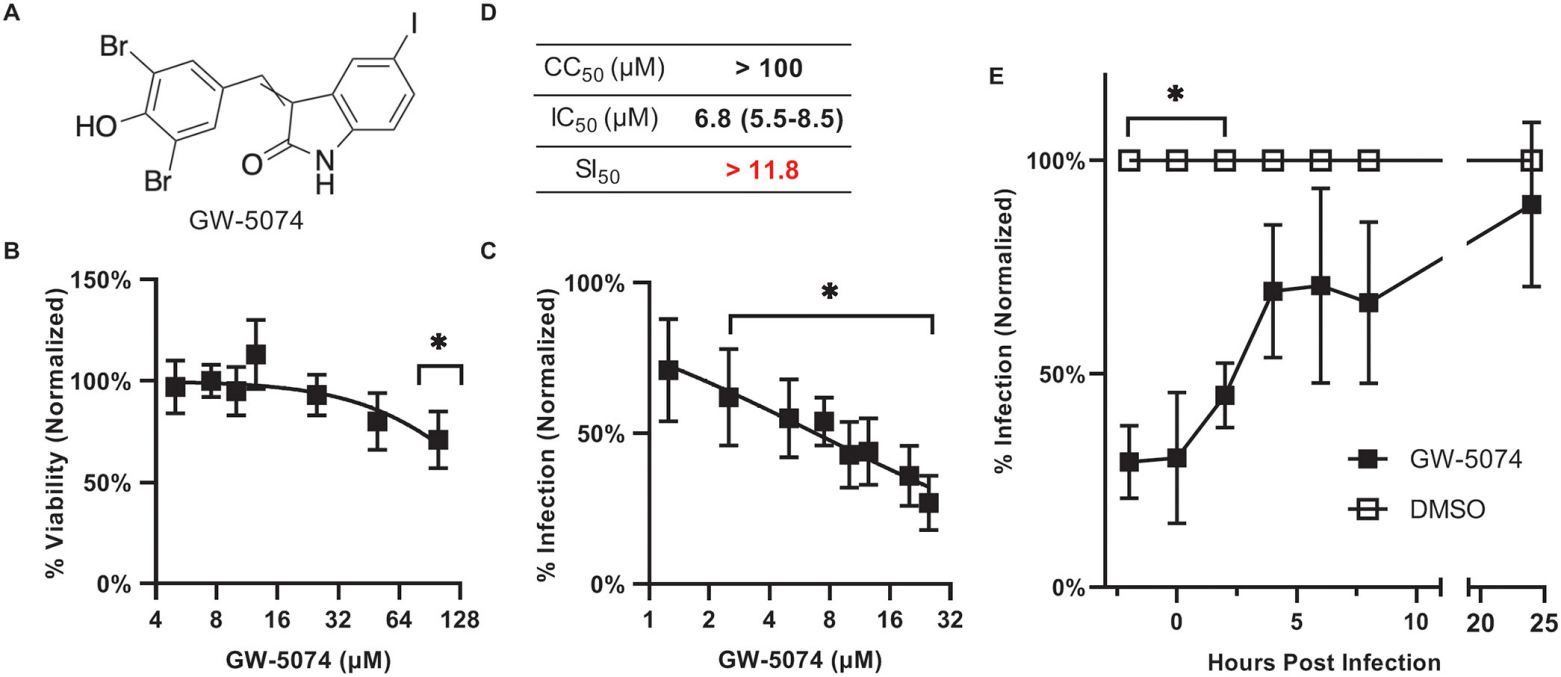
GW-5074 reduces initial JCPyV infection in glial cells. (A) The chemical structure of GW-5074. (B) The toxicity of GW-5074 was evaluated in SVG-A cells after GW-5074 treatment for 72 h. (C) SVG-A cells were pretreated with GW-5074 for 2 h before infection with JCPyV, and cells were maintained in drug-containing media for 72 h. Infection was scored by indirect immunofluorescent quantification of VP1+ cells. (D) The 50% cytotoxic concentration (CC_50_), 50% inhibitory concentration (IC_50_), and 50% selectivity index (SI_50_) are calculated. The 95% confidence interval is specified in parentheses. (E) Early GW-5074 treatment is protective against JCPyV infection. SVG-A cells were treated with GW-5074 or highest concentration vehicle control at various times relative to JCPyV infection. Cells were maintained in drug-containing complete media for 72 h, and infection was scored by indirect immunofluorescent detection of VP1+ cells. Data represent the mean of 3 independent experiments, and error bars represent the standard deviation. Asterisks (*) represent *P* < 0.05 by Student’s *t* Test.

GW-5074 (3-(3,5-dibromo-4-hydroxybenzylidene)-5-iodoindolin-2-one) is a synthetic, disubstituted oxindole (Fig. 1A). GW-5074 was well-tolerated by SVG-A glial cells as no statistically significant difference in GW-5074-associated cytotoxicity was observed at concentrations up to 50 μM (Fig. 1B). To evaluate whether GW-5074 reduces JCPyV infection, we treated SVG-A cells with nontoxic concentrations of GW-5074 for 2 h before infection with JCPyV. GW-5074 treatment reduced initial infection in a dose-dependent manner as evaluated by indirect immunofluorescent quantification of JCPyV VP1+ cells (Fig. 1C). We calculated the 50% inhibitory concentration (IC_50_) to be 6.8 μM (95% confidence interval: 5.5–8.5 μM) using a non-linear dose-response curve (Fig. 1D). These 2 experiments indicate that the minimum 50% selectivity index (SI_50_), defined as the ratio between the 50% cytotoxic concentration (CC_50_) and the IC_50_, associated with GW-5074 treatment, is 11.8. To determine when antiviral activity was lost, SVG-A cells were treated with 20μM GW-5074 or volume-matched vehicle control at various time points relative to infection. GW-5074 significantly inhibited JCPyV infectivity when administered between 2 h before infection and 2 h after infection, with a progressive loss of antiviral activity until 24 h after infection (Fig. 1E).

### GW-5074 reduces viral spread in a multicycle growth assay.

**FIG 2:**
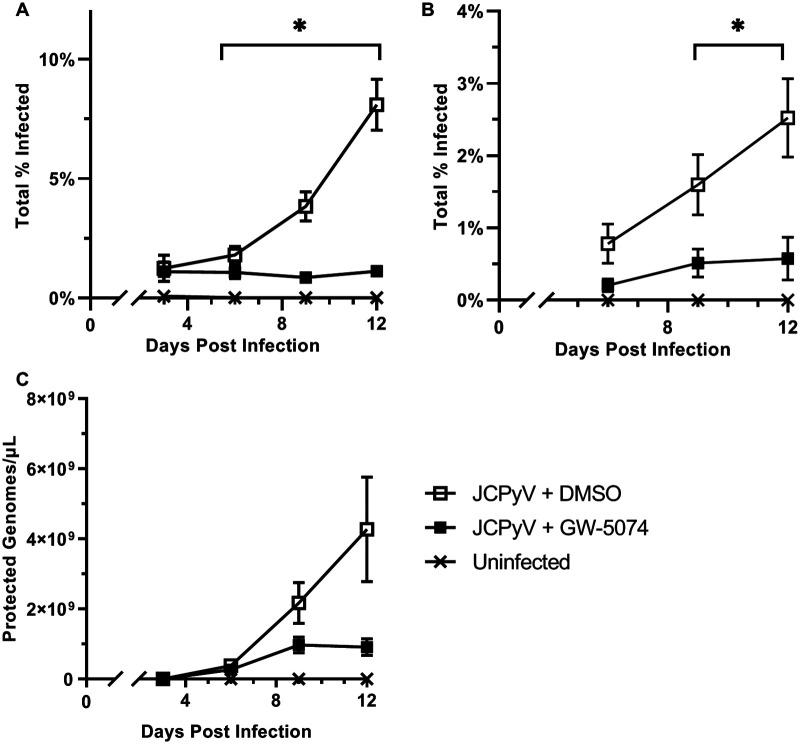
GW-5074 inhibits viral spread. (A) Naive SVG-A cells were infected with JCPyV. At 3 days postinfection (dpi), GW-5074 or volume-matched vehicle control were added. Supplemental doses of GW-5074 were administered every 3 days, and infection was scored by indirect immunofluorescent detection of VP1+ cells every 3 days. (B) Culture media was collected every 3 days and used to reinfect naive SVG-A cells. Infection was scored at 72 h postinfection. (C) To calculate the concentration of infectious virions released by drug-treated and vehicle-treated glial cells, gDNA was isolated from culture media and quantified with a JCPyV-VP2-specific primer/probe set. Data represent the mean of 3 independent experiments, and error bars represent standard deviation. Asterisks (*) represent *P* < 0.05 by Student’s *t* Test.

Because most individuals infected with JCPyV support persistent infection before symptom onset, we asked whether GW-5074 could reduce viral spread in a multicycle growth assay. SVG-A cells were infected with JCPyV for one round of productive infection (3 days) before introducing 20 μM GW-5074 or volume-matched vehicle control. A total of 5 μM GW-5074 or volume-matched vehicle control were then administered every 3 days, and infection was scored via indirect immunofluorescent quantification of VP1+ cells at days 3, 6, 9, and 12 postinfection. GW-5074 treatment significantly inhibited JCPyV infection over time (Fig. 2A). This reduction was most dramatic on day 12, when GW-5074 treatment was associated with an 86% reduction in infection relative to vehicle control. Next, we used a reinfection assay to investigate whether GW-5074 treatment inhibited virion release. Infectious media was collected on days 6, 9, and 12 and used to reinfect SVG-A cells that had not been treated with GW-5074. We observed a significant reduction of the infectivity of media harvested from GW-5074-treated cells relative to that of vehicle-treated cells on days 9 and 12 (Fig. 2B), which suggests that cells treated with GW-5074 released fewer infectious virions. To confirm this, we quantified the concentration of protected viral genomes by qPCR, and found that GW-5074 treatment was associated with a 4.7-fold decrease in protected viral genomes released by day 12 (Fig. 2C). Taken together, these results demonstrate that GW-5074 treatment reduces cell-to-cell spread of JCPyV in an established infection.

### GW-5074 reduces JCPyV infection in normal human astrocytes.

**FIG 3:**
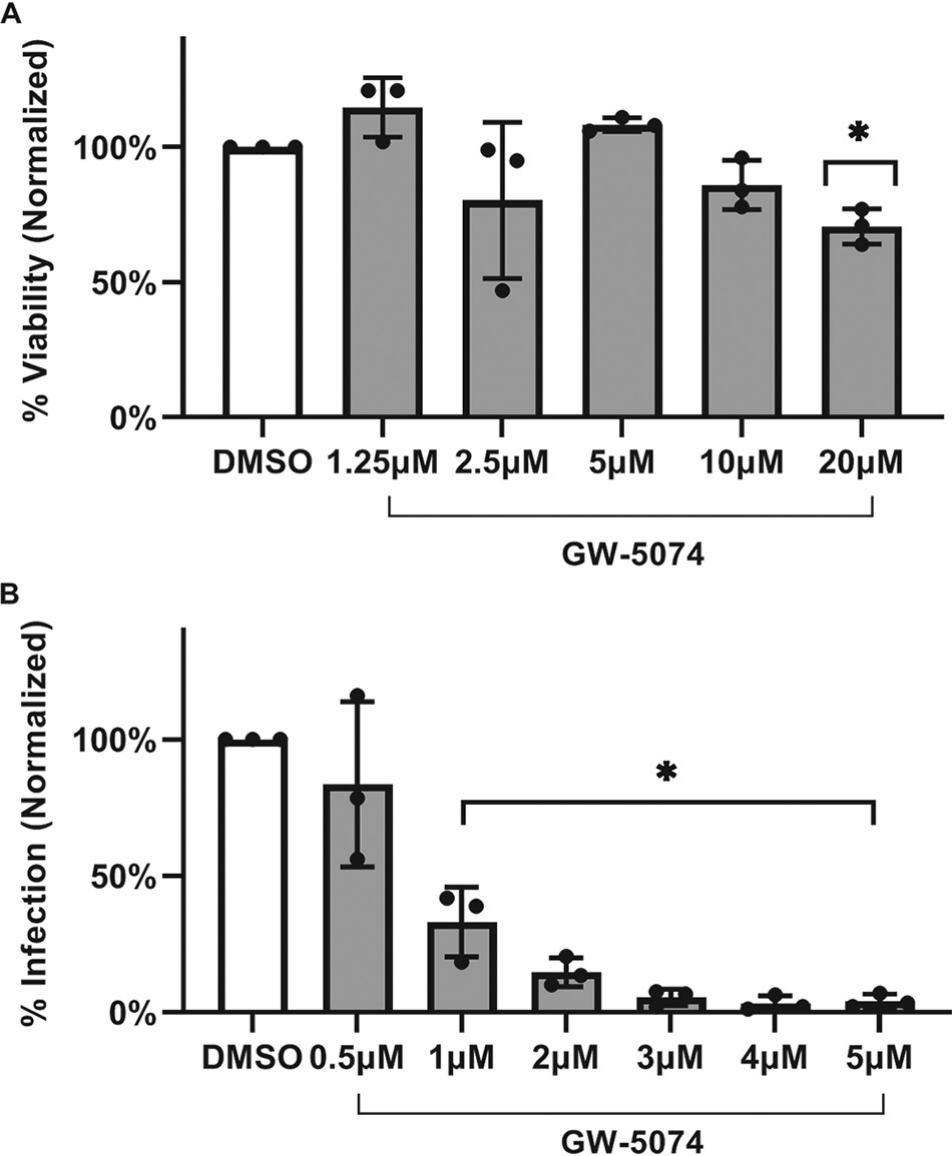
GW-5074 reduces JCPyV infection in normal human astrocytes. (A) The toxicity of GW-5074 was evaluated in normal human astrocytes (NHAs) after GW-5074 treatment for 120 h. (B) NHAs were pretreated with nontoxic doses of GW-5074 for 2 h before infection with JCPyV. Cells were maintained in drug-containing media for 120 h, and infection was scored via indirect immunofluorescent quantification of VP1+ cells. Data represent the mean of 3 independent experiments, and error bars represent the standard deviation. Based on these data, the SI_50_ of GW-5074 in normal human astrocytes is at least 20.2. Asterisks (*) represent *P* < 0.05 by Student’s *t* Test.

Because SVG-A cells are transformed with simian virus 40 (SV40) Large T-Antigen (TAg), we asked whether the antiviral activity of GW-5074 was specific to immortalized cells. We screened GW-5074 in primary normal human astrocytes (NHAs), which are susceptible to JCPyV infection but not transformed with SV40 TAg. We observed that NHA viability was unaffected at GW-5074 concentrations less than 20 μM (Fig. 3A). GW-5074 significantly reduced JCPyV infection in NHAs with an IC_50_ of 0.84 μM (95% confidence interval: 0.70–0.99 μM) (Fig. 3B). Taken together, we calculated the SI_50_ of GW-5074 in normal human astrocytes to be 20.2.

### GW-5074 reduces initial infection by human and simian polyomaviruses.

**FIG 4:**
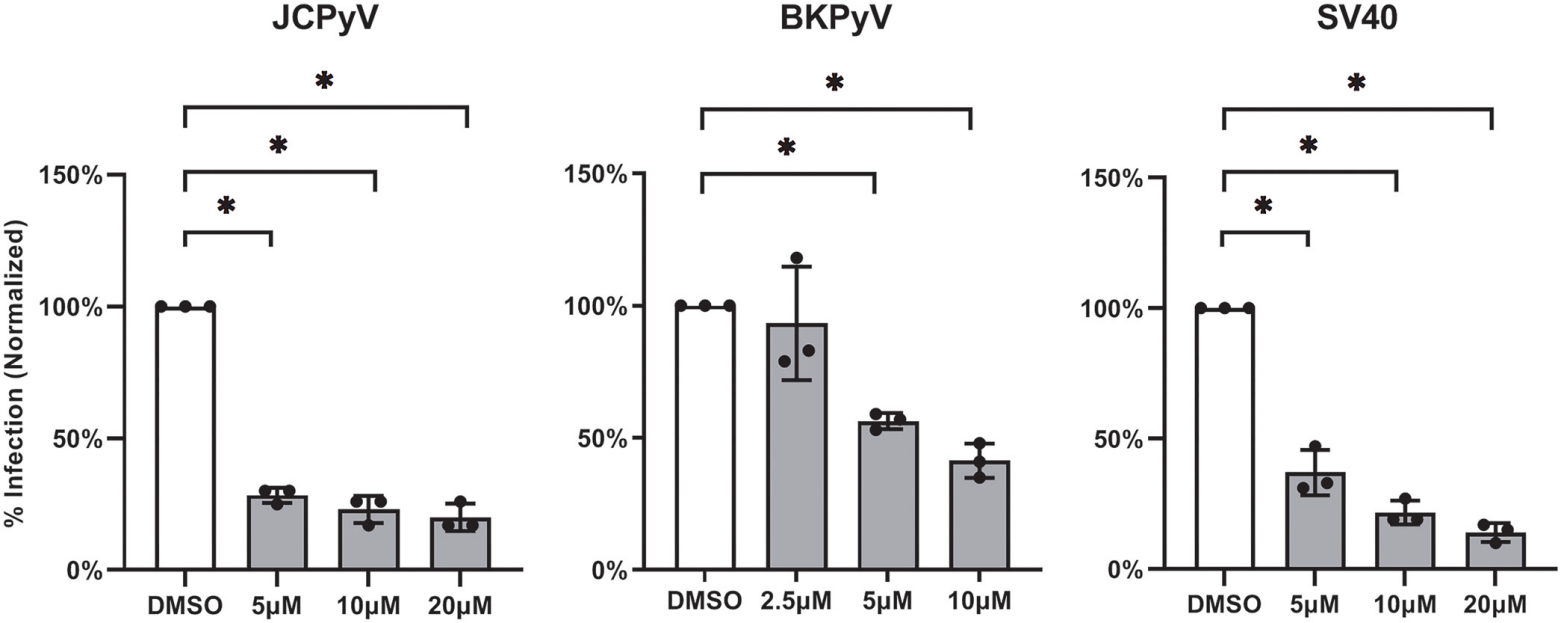
GW-5074 reduces initial infection by human and simian polyomaviruses. SVG-A (JCPyV and SV40) or Vero cells (BKPyV) were pretreated with GW-5074 for 2 h before infection with JCPyV, BKPyV, or SV40. Infection was scored at 72 h postinfection by indirect immunofluorescent detection of VP1+ cells. Asterisks (*) represent *P* < 0.05 by Student’s *t* Test.

We used the human glial cell line, SVG-A, as a host for both JCPyV and SV40, as these cells are susceptible to infection by both viruses. We used the monkey kidney cell line, Vero, as a host for BK Polyomavirus (BKPyV), as BKPyV does not infect SVG-A cells. SVG-A and Vero cells were treated with GW-5074 for 2 h before infection with JCPyV, SV40, or BKPyV to investigate whether the antiviral activity of GW-5074 was specific to JCPyV, or could be extended to other family members. GW-5074 potently reduced infection by JCPyV (maximum percent reduction = 80%), SV40 (maximum percent reduction = 85%), and BKPyV (maximum percent reduction = 59%) (Fig. 4). GW-5074 was nontoxic at concentrations capable of inhibiting infection (Fig. S1).

10.1128/mbio.03583-22.1 FIG S1 Toxicity of GW-5074 in Vero cells. SVG-A and Vero cells were treated with GW-5074 for 72 h, after which cell viability was quantified with the Viral ToxGlo assay (Promega). Data represent the mean of 3 replicates, and error bars represent the standard deviation. Download FIG S1, TIF file, 0.1 MB. Copyright © 2023 Kaiserman et al. 2023 Kaiserman et al. https://creativecommons.org/licenses/by/4.0/ This content is distributed under the terms of the Creative Commons Attribution 4.0 International license.

### GW-5074 inhibits MAPK-ERK signaling events.

**FIG 5:**
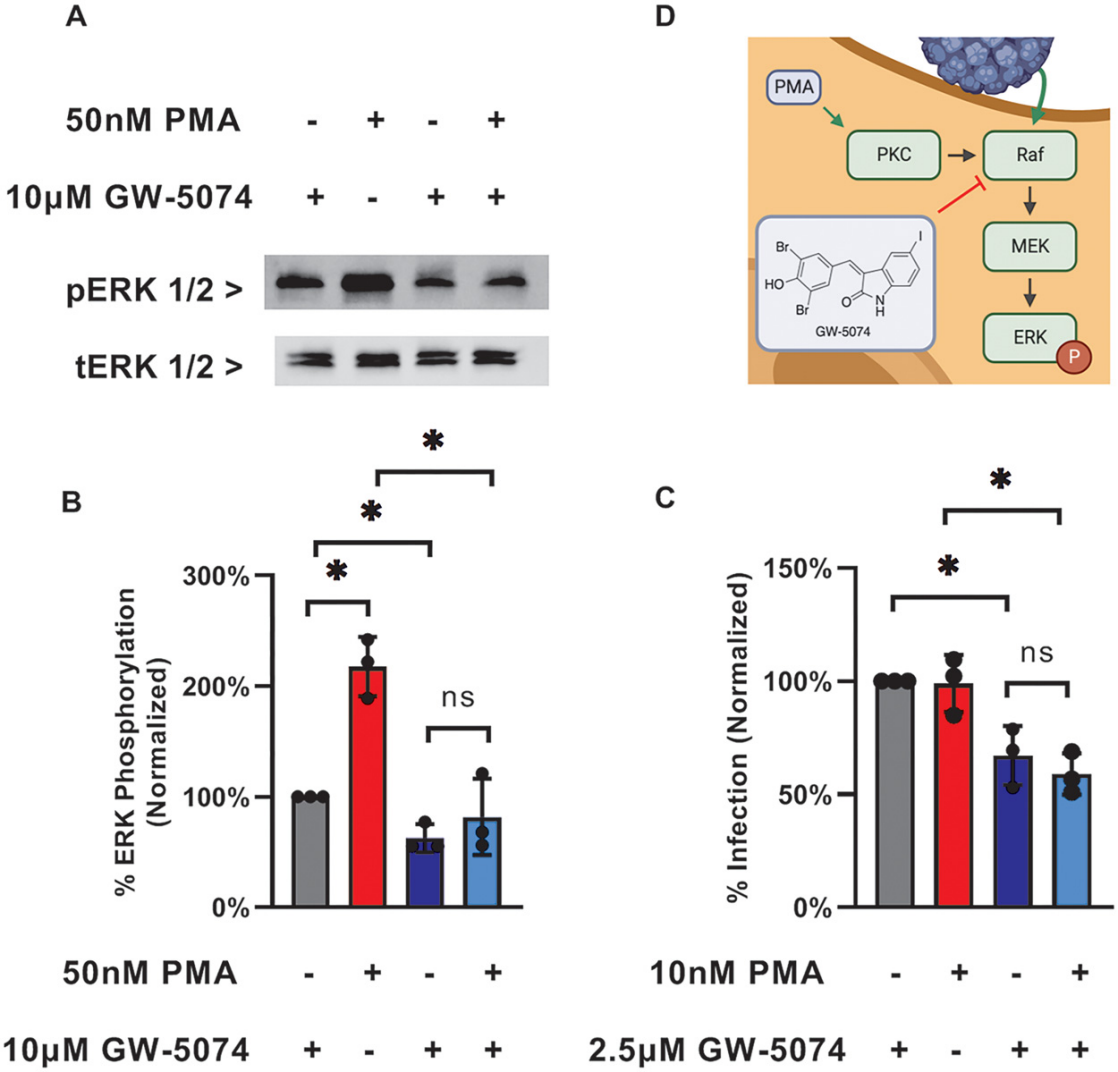
GW-5074 alters JCPyV-induced MAPK-ERK signaling events. (A) GW-5074 inhibits endogenous ERK phosphorylation by the Protein Kinase C agonist PMA. SVG-A cells were pretreated with GW-5074 or highest concentration vehicle control for 3 h. PMA was then administered alone or in the presence of GW-5074 for 1 h. ERK phosphorylation was visualized by Western blot. (B) Quantified results from (A). Data represent the band intensity from 3 independent experiments, and error bars represent the standard deviation. (C) PMA treatment cannot rescue initial JCPyV infection. Data represent the mean of 3 independent experiments, and error bars represent the standard deviation. (D) Proposed mechanism of the anti-JCPyV activity of GW-5074. GW-5074 is known to inhibit C-Raf, and these data suggest that GW-5074 treatment reduces viral infection by antagonizing MAPK-ERK signaling (19). PKC = Protein Kinase C. Image created with BioRender.com. Asterisks (*) represent *P* < 0.05 by Student’s *t* Test.

GW-5074 is known to inhibit C-Raf, a MAP kinase kinase kinase, so we hypothesized that the anti-JCPyV activity of GW-5074 could be attributed its antagonistic effect on MAPK-ERK signaling. We found that GW-5074 significantly reduced endogenous ERK activation relative to volume-matched vehicle control (Fig. 5A and 5B). We also treated SVG-A cells with the Protein Kinase C agonist phorbol 12-myristate 13-acetate (PMA), which directly activates C-Raf, in the presence or absence of GW-5074. ERK activation could not be restored when cells were incubated with PMA and GW-5074 together (Fig. 5A and 5B). We then investigated this signaling antagonism in the context of JCPyV infection by attempting to rescue virus infection with PMA treatment. No significant difference was observed in JCPyV infection in cells treated with GW-5074 and PMA (Fig. 5C).

## DISCUSSION

The oxindole GW-5074 3-(3,5-dibromo-4-hydroxybenzylidene)-5-iodoindolin-2-one is a commercially available MAPK-ERK inhibitor first synthesized by Lackey et al. (2000) to inhibit C-Raf, a MAP kinase kinase kinase (19). In initial screening experiments, we found that GW-5074 potently inhibited JCPyV infection at nontoxic concentrations in SVG-A glial cells with a 50% inhibitory concentration of 6.8 μM (95% confidence interval = 5.5–8.5 μM) (Fig. 1B and 1C). To estimate the therapeutic potential of GW-5074, we relied on the 50% selectivity index (SI_50_), which is defined as the ratio between the 50% cytotoxic concentration (CC_50_) and the 50% inhibitory concentration (IC_50_). Higher SI_50_ values are associated with more promising therapies, as these compounds have a larger window between concentrations associated with effective response and concentrations associated with cytotoxicity. SI_50_ > 10 is often considered an effective metric for drug development (8).

Using the upper end of the 95% confidence interval, we calculated the minimum SI_50_ associated with GW-5074 treatment in SVG-A cells to be 11.8 (Fig. 1D). Importantly, we also showed that GW-5074 inhibits JCPyV infection in normal human astrocytes with an IC_50_ of 0.84 μM (95% confidence interval = 0.70–0.99 μM), and we calculated the minimum SI_50_ of GW-5074 in these primary cells to be 20.2 (Fig. 3B). That the IC_50_ of GW-5074 in primary astrocytes is over 8 times smaller than the IC_50_ of GW-5074 in the transformed SVG-A glial cells suggests that GW-5074 could be effective *in vivo*, because lower concentrations of drug would be needed to exert an antiviral effect in primary cells. In both cell types, the SI_50_ values are above the recommended threshold, and exceed the SI_50_s of many established anti-JCPyV compounds, including chlorpromazine (SI_50_ = 2.0), mefloquine (SI_50_ = 4.0), and Retro-2^cycl^ (SI_50_ = 7.4) (8).

Because patients are persistently infected with JCPyV before PML onset, we asked whether GW-5074 could eliminate the spread of an established viral infection. We infected untreated SVG-A cells for 1 infectious cycle (3 days) before introducing GW-5074 or volume-matched vehicle control with supplemental doses every 3 days. A significant decrease in JCPyV infection was observed by 6 days postinfection (dpi), and this effect was most striking at 12 dpi, when we observed an 86% reduction in infection in GW-5074-treated cells relative to vehicle-treated cells (Fig. 2A). We also used indirect and direct methods to determine that GW-5074-treated cells released fewer infectious virions than vehicle-treated cells. The supernatant collected from GW-5074- and vehicle-treated cells was first incubated with naive SVG-A cells in a reinfection assay. We observed significant reductions in the infectivity of the supernatant from GW-5074-treated cells relative to that of vehicle-treated cells by 9 dpi (Fig. 2B). We then used qPCR to calculate a near 5-fold reduction in the concentration of protected viral genomes in the supernatant of GW-5074-treated cells when compared to the supernatant of vehicle-treated cells (Fig. 2C). Taken together, these data suggest that GW-5074 can inhibit virus spread in an established infection, which is necessary for the *in vivo* treatment or prevention of PML.

That GW-5074 inhibits BKPyV infection suggests that GW-5074 could be used to treat diseases, including BKPyV-associated nephropathy and hemorrhagic cystitis (Fig. 4 and Fig. S1) (8, 16–18). However, more research is needed to determine the therapeutic potential of this compound in primary renal or bladder epithelia and to measure the ability of GW-5074 to inhibit BKPyV spread.

Because GW-5074 is known to inhibit C-Raf, we suspected that GW-5074 may reduce JCPyV infection by antagonizing MAPK-ERK signaling (19). GW-5074 significantly reduced JCPyV infection when administered between 2 h before infection and 4 h after infection, with a progressive loss of antiviral activity until 24 h after infection (Fig. 1E). These kinetics are consistent with previous reports of JCPyV-induced ERK phosphorylation in SVG-A cells (20–22). ERK phosphorylation occurs immediately upon virion internalization, peaks at 2 h post internalization, and decreases until 9 h post internalization; later ERK phosphorylation is observed, which could facilitate late-stage events in the viral life cycle (20–22). We also observed a ~50% decrease in ERK phosphorylation in GW-5074-treated SVG-A cells relative to vehicle-treated SVG-A cells (Fig. 5A and B). Treatment with the Protein Kinase C (PKC) agonist PMA failed to induce endogenous ERK phosphorylation in GW-5074-treated cells, which suggests that GW-5074 inhibits MAPK-ERK signaling downstream of PKC (Fig. 5A and B). This is consistent with previous reports demonstrating that PKC directly activates C-Raf (23). To ensure that this signaling antagonism was relevant in JCPyV infection, we attempted to rescue virus infection with prolonged PMA treatment. However, there was no statistically significant difference in JCPyV infection in SVG-A cells treated with GW-5074 relative to cells treated with both GW-5074 and PMA (Fig. 5C). These data ultimately suggest that GW-5074 irreversibly inhibits the MAPK-ERK signaling events necessary to establish a productive viral infection by inhibiting C-Raf (Fig. 5D).

However, these data also reveal that GW-5074 could possess a second mechanism of antiviral activity, depending on the cellular context. While JCPyV is known to require virus-induced activation of the MAPK-ERK pathway in the SV40 Large T-Antigen (TAg) transformed SVG-A cell line, the PI3K/AKT/mTOR pathway seems to dominate in primary normal human astrocytes (20–22, 24–27). These signaling cascades involve distinct kinases but exhibit significant cross talk via cross-inhibition and cross-activation (28). Because of the distinct signaling events necessary to establish a productive viral infection, different inhibitors seem to be more effective in different target cells. MAPK-ERK inhibitors like U0126 (MEK1/2), PD98059 (MEK1), and sorafenib (C-Raf, receptor tyrosine kinase) inhibit JCPyV infection in SVG-A glial cells (21, 24). In contrast, in primary normal human astrocytes, inhibitors of PI3K, AKT, and mTOR, including wortmannin (PI3K), MK2206 (AKT), and rapamycin (mTOR), inhibit JCPyV infection while MAPK-ERK inhibitors like U0126 fail to reduce infection (25). That GW-5074 potently reduces JCPyV infection in normal human astrocytes therefore suggests that GW-5074 could exert antiviral activity through another compensatory mechanism in this cell type (Fig. 3B). Furthermore, while JCPyV requires MAPK-ERK signaling to infect target cells, the related simian polyomavirus SV40 does not require MAPK-ERK signaling for infection (21, 26). We, therefore, hypothesized that GW-5074 would not inhibit SV40 infection but observed that GW-5074 reduces initial infection by JCPyV, BKPyV, and SV40 (Fig. 4). Recent reports have shown that GW-5074 also inhibits nuclear transport by disrupting the importin α/β_1_ heterodimer, and virus-like JCPyV particles fail to enter the nucleus when either importin α or β is not present (29–32). It is possible that GW-5074 could bind to both C-Raf and the importin α/β_1_ heterodimer to exert antiviral activity through 2 mechanisms, and this complexity requires further investigation.

This report also has some limitations. The use of GW-5074 to treat PML requires further research concerning its pharmacokinetics and pharmacodynamics, which these data do not address. In particular, *in silico* and *in vitro* approaches must be used to explore if GW-5074 could cross the barriers that restrict access to the CNS. Diversity-oriented synthesis studies should also generate GW-5074 analogues to increase the SI_50_ values and to optimize CNS permeability. Despite these limitations, we believe that this report addresses unmet needs in drug development for PML by identifying a candidate compound that could succeed *in vivo.*

## MATERIALS AND METHODS

### Cells and viruses.

SVG-A glial cells were maintained in Minimum Essential Media ([MEM], Corning) supplemented with 10% fetal bovine serum (Atlanta Biologics) and 1% Antibiotic-Antimycotic (ThermoFisher) (33). Vero cells were maintained in MEM supplemented with 5% fetal bovine serum and 1% Antibiotic-Antimycotic. Primary normal human astrocytes were maintained in normal human astrocyte media (ScienceCell). Cells were kept in an incubator at 37°C and 5% CO_2_. The Mad1-SVE-delta strain of JCPyV (Turbo), the 777 strain of SV40, and the Dunlop strain of BKPyV were used for infection assays; all have been described previously (34).

### Compounds.

GW-5074 was purchased from Tocris. PMA was purchased from Sigma-Aldrich. Compounds were reconstituted in DMSO (Sigma-Aldrich), and used at the indicated concentrations. Compound stocks and solutions were handled according to the distributor’s protocol.

### Toxicity assays.

Cells were seeded into tissue culture-treated 96-well plates (Corning) at a density of 5,000 cells/well. The next day, cells were treated with indicated concentrations of drug or volume-matched vehicle control in phenol-free complete media. Seventy-two hours after treatment, viability was determined by visual inspection and analysis with the Viral ToxGlo (Promega) assay, according to the manufacturer’s protocol. The Viral ToxGlo assay quantifies cytopathic effects with a luminescent signal that is directly proportional to the amount of ATP present. Luciferase activity was analyzed with a Glomax Multidetection System (Promega) after incubation of cells with the Viral ToxGlo ATP Detection Reagent for 10 min in the dark.

### Infection assays.

For initial infections, SVG-A cells were seeded into tissue culture-treated 96-well plates (Corning) at a density of 5,000 cells/well. The next day, cells were treated with drug or volume-matched vehicle control for 2 h in complete media at 37°C, 5% CO_2_. Cells were then washed with 1 × phosphate-buffered saline (PBS) and incubated with JCPyV, BKPyV, or SV40 at a multiplicity of infection (MOI) of 5 ffu/cell in serum-free media containing drug or volume-matched vehicle control at 37°C, 5% CO_2_. After allowing internalization of virions for 2 h, infectious media was aspirated, unbound virus was washed out with 1 × phosphate-buffered saline PBS, and cells were given complete media containing drug or volume-matched vehicle control. Cells were then maintained at 37°C, 5% CO_2_ until the specified time points. For the time course, cells were either pretreated with 20 μM GW-5074 or volume-matched vehicle control reconstituted in complete media, or maintained in complete media without drug or vehicle until the specified time point, when complete media was aspirated and complete media containing 20 μM GW-5074 or volume-matched vehicle control was introduced. Infection was performed using the aforementioned protocol with a MOI = 5 ffu/cell and scored at 72 h postinfection via indirect immunofluorescent quantification of VP1+ cells. For viral spread assays, SVG-A cells were seeded into tissue culture-treated 96-well plates (Corning) at a density of 2,500 cells/well. The next day, complete media was aspirated, cells were washed with 1 × PBS, and incubated with serum-free media containing JCPyV (MOI = 5 ffu/cell) for 2 h at 37°C, 5% CO_2_. Cells were then washed with 1 × PBS and incubated with complete media for 3 days after infection at 37°C, 5% CO_2_. Wells were then topped with 100 μL complete media to reach a final concentration of 20 μM GW-5074, or volume-matched vehicle control. Every 3 days, wells were supplemented with 50 μL complete media containing 5 μM GW-5074 or volume-matched vehicle control. Infection was scored via indirect immunofluorescent quantification of VP1+ cells at days 3, 6, 9, and 12 postinfection. Reinfections were performed by incubating untreated SVG-A cells (seeded into tissue culture-treated 96-well plates at a density of 5,000 cells/well) with 35 μL of supernatant from each treatment group in the multicycle growth assay for 2 h at 37°C, 5% CO_2_. Supernatant was then washed out with 1 × PBS and replaced with complete media for 72 h when infection was scored. For infections requiring serum starvation (Fig. 5), infections were performed with CsCl-purified JCPyV (MOI = 5 ffu/cell). Virus purification was accomplished according to established methods (35). All other experiments were performed using non-purified viral lysate. For experiments with normal human astrocytes, cells were seeded into tissue culture-treated 96-well plates (Corning) at a density of 10,000 cells/well. After pretreatment and virion internalization, cells were maintained in drug-containing media for 5 days after infection.

### Indirect immunofluorescence.

To score infections at indicated time points, complete media was aspirated, and cells were fixed in ice-cold methanol for at least 20 min at −20°C. Cells were rehydrated with 1 × PBS, further permeabilized with 0.1% Triton-X for 10 min at room temperature, and blocked with 5% goat serum (ThermoFisher) and 0.1% Triton-X (USB) in 1 × PBS for 1 h at room temperature. Cells were incubated with the VP1-specific primary antibody PAb597 in 1 × PBS (diluted 1:50) overnight at 4°C, washed extensively with 1 × PBS, and then incubated with goat anti-mouse Alexa Fluor 488 conjugated antibody (ThermoFisher) in 1 × PBS (diluted 1:1,000) for 1 h at room temperature in the dark. Finally, cells were counterstained with 4′,6-diamidino-2-phenylindole (DAPI) in 1 × PBS (diluted 1:1000) for 5 min at room temperature, and washed again in 1 × PBS prior to imaging. The 20x objective of a Ti2-E inverted fluorescence microscope (Nikon) was used to analyze cells for nuclear VP1 staining and calculate total cell number. Cell count analysis was performed using Elements High Content imaging software (Nikon). PAb597 was generated against SV40 VP1 but also cross-reacts with BKPyV VP1 and JCPyV VP1, which enables the efficient scoring of human and simian polyomavirus infection with 1 primary antibody. The PAb957 hybridoma was a generous gift from Ed Harlow.

### Quantification of protected genomes by qPCR.

The supernatant from each treatment group in the multicycle growth assay was frozen at −20°C before qPCR quantification of protected viral genome content. A total of 100 μL of sample was first treated with DNase 1 (New England Biolabs) for 30 min at 37°C, followed by a 10-minute inactivation at 75°C, according to the manufacturer’s protocol. Pretreatment with DNase removes non-encapsidated and contaminating DNA. Samples were next processed using a DNeasy blood and tissue kit (Qiagen) to isolate genomic DNA. Quantitative PCR was conducted using VP2 TaqMan Assay primer/probe set (probe: /5HEX/TGTTCTCCA/ZEN/CAATCTCCCAGGCTT/3IABkFQ, primer 1: CCTGGAGTGAATGCCTTTGT, primer 2: AGAGGTTAAGGCTGGCAAATC) (IDT), and run on a Bio-Rad CFX96 detection system using the CFX Prime Time qPCR Protocol with 35 cycles. Mad1-pBR322 plasmid was used in a serial dilution to generate a standard curve of JCPyV DNA, and copy number calculations were made using the ThermoFisher copy number tool.

### Western blotting.

Cells were lysed on ice with Pierce radioimmunoprecipitation assay (RIPA) containing complete EDTA-free protease inhibitor cocktail (Sigma/Roche). Protein concentration was determined with the Pierce Rapid Gold BCA Kit (ThermoFisher) according to the manufacturer’s protocol. Western blot samples were prepared with 4x laemmli buffer (Bio-Rad) under reducing conditions, and boiled for 10 min. Samples were loaded into 4% to 15% gradient Mini-Protean TGX Stain-Free precast gels (Bio-Rad), and Stain-Free gels were run initially at 60V for 10 min then 120V for 50 min with a PowerPac (Bio-Rad). Total protein content per lane was determined by activating and imaging the Stain-Free gel on a ChemiDoc MP imaging system (Bio-Rad), and then transferred to a 0.2μm-pore-size nitrocellulose membrane (Bio-Rad) via the semidry transfer method. Stain-Free gels were reimaged after transfer to ensure complete protein transfer onto the membrane. pERK was visualized with CST9101, and tERK was visualized with CST9102 (Cell Signaling Technologies, diluted 1:1,000 in 1% casein buffer/Tris-buffered saline (TBS), and incubated overnight at 4°C). Blots were washed 3 times with 1 × TBS-T then incubated with goat anti-mouse horseradish peroxidase (ThermoFisher, diluted 1:10,000 in 1 × TBS-T) secondary antibodies at room temperature for 1 h. Blots were washed 3 more times with TBS-T then incubated with ClarityMax (Bio-Rad) according to the manufacturer’s protocol. Blots were imaged on a ChemiDoc MP imaging system (Bio-Rad), and band intensity was then quantified with Image Lab (Bio-Rad) and normalized to volume-matched vehicle control.

### ERK phosphorylation and rescue.

SVG-A cells were serum-starved for 48 h prior to ERK phosphorylation experiments to reduce initial pERK levels. For Fig. 5A, cells were treated with 10 μM GW-5074 reconstituted in serum-free culture media for 3 h, then 50 nM PMA was added for 1 h. Cells were lysed with RIPA and ERK phosphorylation was visualized by the aforementioned Western blot protocol. For Fig. 7C, 2.5 μM GW-5074 reconstituted in serum-free media was administered to SVG-A cells for 2 h at 37C, 5% CO_2_. Then, cells were infected with JCPyV for 2 h according to the above infection protocol. At the same time, 10 nM PMA reconstituted in serum-free media was introduced to relevant wells. After infection, serum-free media containing JCPyV was aspirated, and cells were incubated with serum-free media containing PMA alone, GW-5074 alone, or a combination treatment containing GW-5074 and PMA for 1 h. PMA was then removed by aspirating media, washing cells with serum-free media, and introducing serum-free media containing just GW-5074 or volume-matched vehicle control. Serum-free media containing drug or volume-matched vehicle control was replaced with complete media containing GW-5074 or volume-matched vehicle control 6 h after infection. Infection was scored via indirect immunofluorescent quantification of VP1+ cells 96 h postinfection.

### Pharmacological parameters.

Non-linear curves were calculated and fit using GraphPad Prism. The 95% confidence intervals for the 50% inhibitory concentration (IC_50_) were also calculated with GraphPad Prism. The 50% selectivity index (SI_50_) is defined as the ratio between the 50% cytotoxic concentration (CC_50_) and the IC_50_.

### Statistical analysis.

Data represent the average of 3 independent experiments performed in triplicate, and error bars represent the standard deviation. Statistical significance was determined using a two-tailed, unpaired Student’s T Test with Welch’s Correction for unequal variances. The cutoff for statistical significance was *P* < 0.05, and is indicated with an asterisk. Statistical analyses were performed GraphPad Prism. Normalization to vehicle control is indicated when relevant. Points on bar graphs indicate means of individual experiments performed in triplicate.

### Data availability.

All data associated with this report are presented in the paper or the Supplementary Materials.
