# Supplementary figures and images for: The Oxindole GW-5074 Inhibits JC Polyomavirus Infection and Spread by Antagonizing the MAPK-ERK Signaling Pathway
Source: mBio. 2023 Feb 14;14(2):e03583-22. doi: 10.1128/mbio.03583-22 (PMC10127638; doi:10.1128/mbio.03583-22)

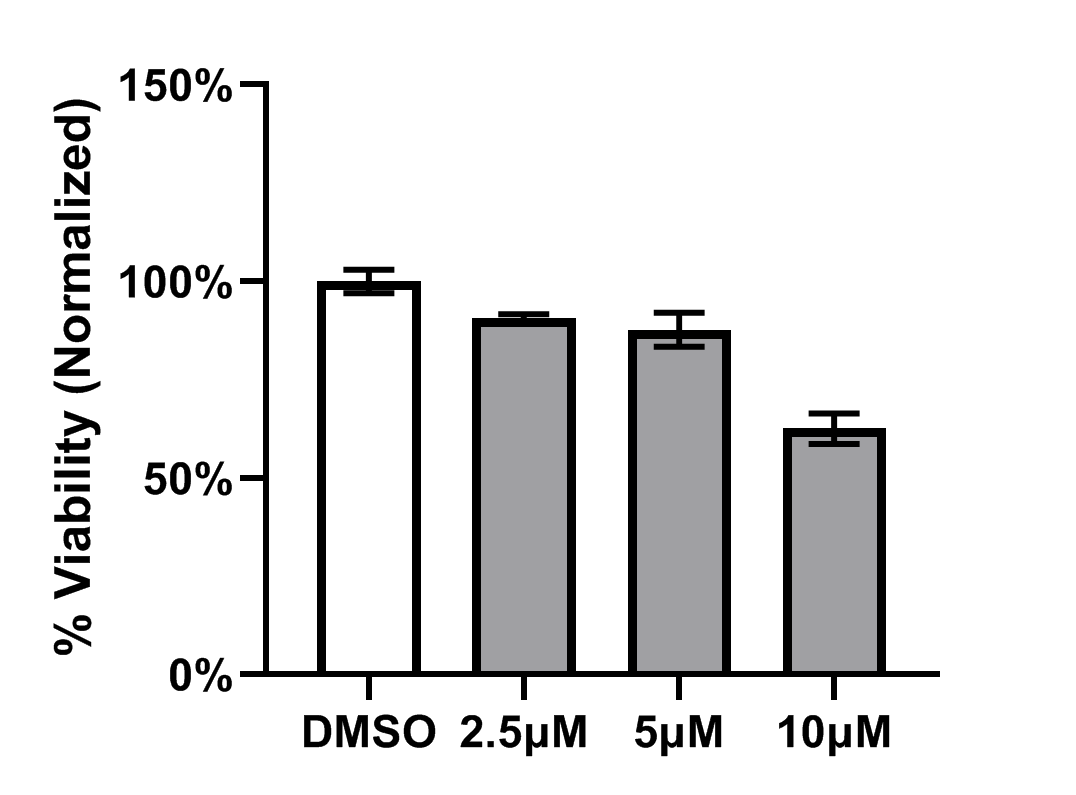

Supplement: FIG S1 [file mbio.03583-22-s0001.tif]
